# Cordillera Zealandia: A Mesozoic arc flare-up on the palaeo-Pacific Gondwana Margin

**DOI:** 10.1038/s41598-017-00347-w

**Published:** 2017-03-21

**Authors:** L. A. Milan, N. R. Daczko, G. L. Clarke

**Affiliations:** 1 Earth Sciences, School of Environmental and Rural Science, The University of New England, Armidale, NSW 2351 Australia; 2 Australian Research Council Centre of Excellence for Core to Crust Fluid Systems (CCFS) and GEMOC, Department of Earth and Planetary Sciences, Macquarie University, Armidale, NSW 2109 Australia; 3 School of Geosciences, The University of Sydney, Sydney, NSW 2006 Australia

## Abstract

Two geochemically and temporally distinct components of the Mesozoic Zealandia Cordilleran arc indicate a shift from low to high Sr/Y whole rock ratios at c. 130 Ma. Recent mapping and a reappraisal of published Sr-Nd data combined with new *in-situ* zircon Hf isotope analyses supports a genetic relationship between the two arc components. A reappraisal of geophysical, geochemical and P-T estimates demonstrates a doubling in thickness of the arc to at least 80 km at c. 130 Ma. Contemporaneously, magmatic addition rates shifted from ~14 km^3^/my per km of arc to a flare-up involving ~100 km^3^/my per km of arc. Excursions in Sr-Nd-Hf isotopic ratios of flare-up rocks highlight the importance of crust-dominated sources. This pattern mimics Cordilleran arcs of the Americas and highlights the importance of processes occurring in the upper continental plates of subduction systems that are incompletely reconciled with secular models for continental crustal growth.

## Introduction

Cordilleran arcs form within continental crust above convergent plate margins as trench-parallel belts of voluminous calc-alkaline magmatism. They are important locations for continental crustal growth^1^, with the bulk of the igneous rocks being emplaced in episodes of short (5–20 my) duration high magmatic flux, called magmatic flare-ups that have a periodicity of 30–70 my^2–8^. The production of such large volumes of intermediate to felsic melt requires ~50% contribution of partially melted mafic lower continental crust and lithospheric mantle, and ~50% from melt sourced from the mantle wedge above the subducting oceanic slab^5, 7–11^. As the ratio of plutonic to volcanic rock is typically 30/1 or higher, plutonism is the dominant expression of a flare-up event^12^. A dense root of ultramafic residues/cumulates is produced and eventually delaminates and founders into the mantle^13–16^, possibly balancing the difference between dominantly basaltic island arc crust and the average andesitic composition of continental crust^1, 17, 18^. Long-lived Cordilleran arcs record repetitive thickening via under-thrusting and shortening of the foreland, with partial melting of this fertile crust facilitating the magmatic flare-up event (75–100 km^3^/my per km of arc). Cyclicity is recorded by repeated short-lived (5–20 my) high volume magmatic flux events involving magmas with high Sr/Y ratios and isotopic excursions to evolved signatures that punctuate background “steady state” magmatic flux rates less than 30 ± 10 km^3^/my per km of arc involving magmas with low Sr/Y ratios^7, 12, 19^. It is posited that the removal of the residue/cumulate root through foundering induces a new cycle^5^. The ensuing influx of asthenosphere returns the arc to background levels of flux and juvenile isotopic compositions.

In this contribution, we utilise 1:250,000 scale mapping^20^, geochemistry and geochronology^21–30^ to identify a flare-up event in a 200 km long segment of a Mesozoic arc formed along the Pacific margin of Gondwana (Cordillera Zealandia: a title introduced by A. Tulloch in conference presentations). Two contrasting models proposed for this segment of the arc involve allochthonous^29, 31–33^ or autochthonous^34–36^ older (>c. 130 Ma) arc material. New *in-situ* zircon Hf isotope data and a reappraisal of published Sr-Nd-Hf data support a relationship between the older and younger arc components.

## Geological Setting

**Figure 1:**
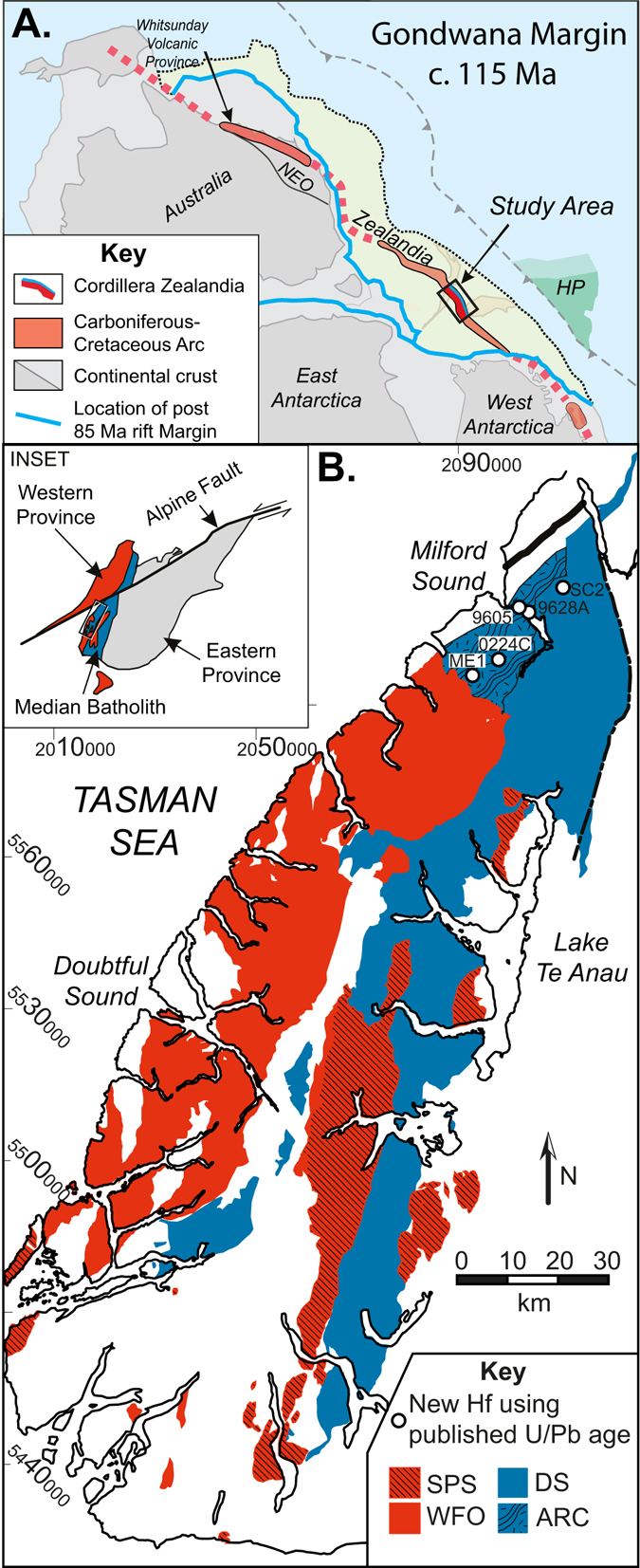
(**A**) Tectonic reconstruction of the Palaeo-Pacific Gondwana Margin at c. 115 Ma (modified from Mortimer,^73^). The Whitsunday Volcanic Province has been viewed as a correlative to the Cretaceous Flare up in New Zealand^74^, however some features have been interpreted as a large igneous province^75^. NEO = New England Orogen. The yellow shaded region represents the Zealandia orogenic belt prior to rifting and fragmentation at 85 Ma. HP = Hikurangi Plateau (approximate location). (**B**) Geological map of New Zealand showing main plutonic suites; WFO (Western Fiordland Orthogneiss), SPS (Separation Point Suite) and DS (Darran Suite). The ARC (Arthur River Complex) is comprised of an older Carboniferous magmatic component and, and a younger Jurassic to Cretaceous magmatic component that overlaps with the Darren Suite in age^25, 53, 54, 76^. This study includes Cretaceous ARC samples that have not been formally assigned to a suite however are considered a correlative of the Darren Suite based on age and geochemistry^25, 53, 54^. A further sample defined as Darren Suite is also included. Modified from Turnbull *et al.* ^20^, available under CC BY 4.0 license (http://creativecommons.org/licenses/by/4.0/). Inset: Palinspastic reconstruction of Median Batholith prior to dextral displacement along Alpine Fault.

New Zealand comprises Permian to Cretaceous forearc terranes (Eastern Province) that had accreted to the Pacific margin of Gondwana (Western Province) by the Triassic (Fig. 1). Both provinces are intruded by the Median Batholith (>10,000 km^2^), which resulted from episodic Cambrian to Early Cretaceous (500–105 Ma) subduction-related plutonism^21, 22, 37^.

**Figure 2:**
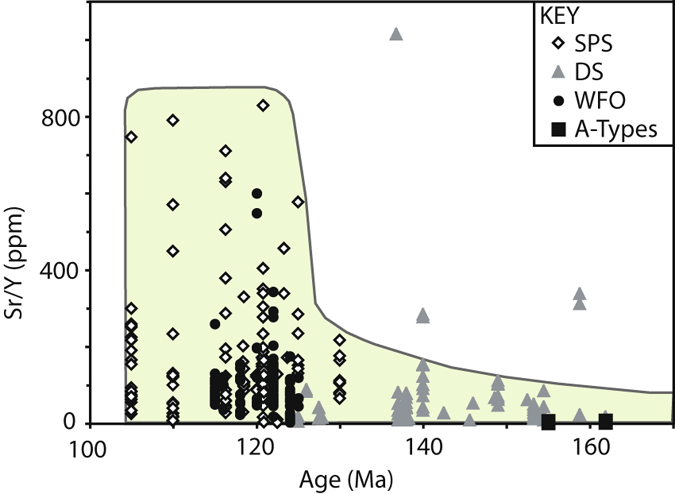
Sr/Y (ppm) over time displays the shift to a garnet rich, plagioclase free source at <c. 130 Ma (Geochemical data from Allibone *et al.* ^21, 22^ and references therein).

There are three volumetrically important Mesozoic components of the Median Batholith (Fig. 1): (i) Triassic–Early Cretaceous (>129 Ma) calc-alkaline rocks of the Darran Suite characterised by dominantly low Sr/Y ratios (Fig. 2; defined as less than 40^38^); (ii) Early Cretaceous (<125 Ma) alkaline-calcic granitoid of the Separation Point Suite; and (iii) Early Cretaceous (<125 Ma) pyroxene ± hornblende diorite and monzodiorite of the Western Fiordland Orthogneiss. Both the Separation Point Suite and Western Fiordland Orthogneiss are characterised by dominantly high Sr/Y ratios and represent upper and lower crustal components of the flare-up event respectively (Fig. 2) ^21–23, 32, 36, 39^.

## Crustal Profile

**Figure 3:**
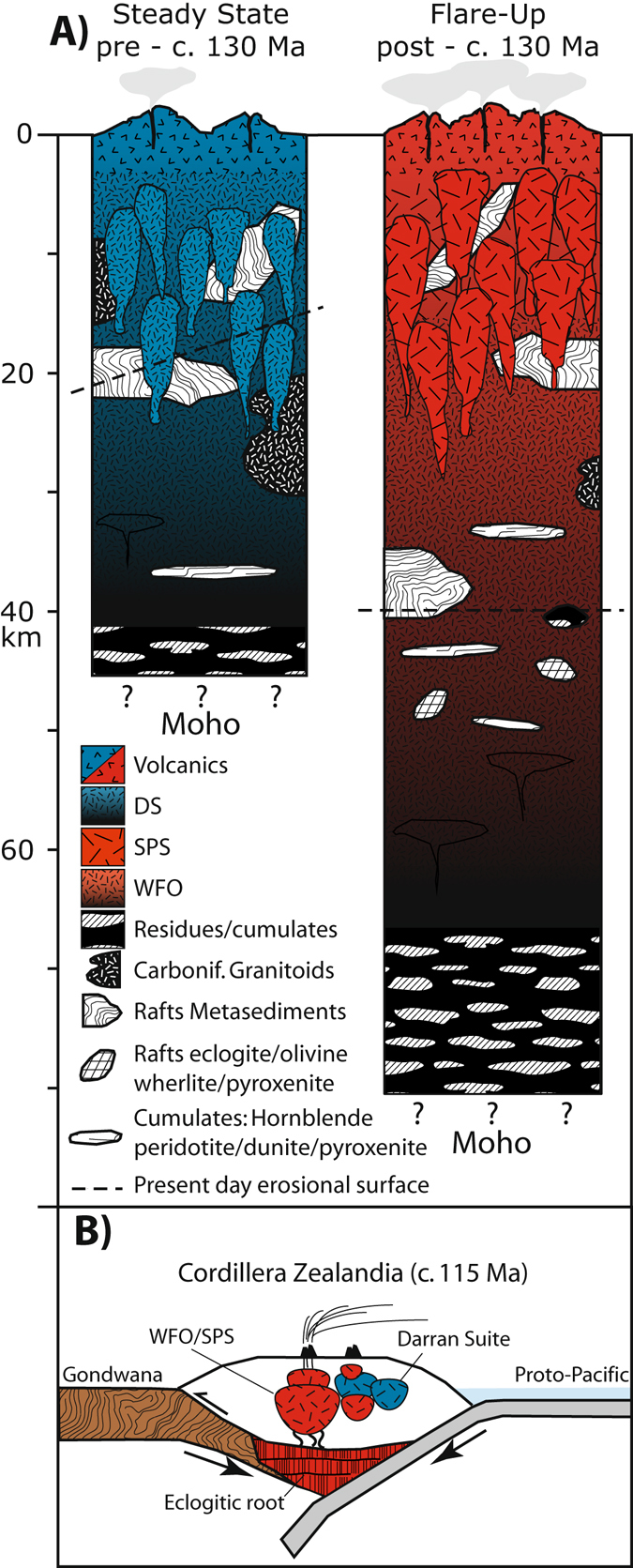
(**A**) Estimated crustal profile of the magmatic arc before and after c. 130 Ma. See Fig. 1. for abbreviations. (source data: Tulloch and Challis^44^; Eberhart-Phillips and Reyners^45^; Scott, *et al.* ^29^; De Paoli *et al.* ^47^. Figure adapted from Tibaldi *et al.* ^77^). (**B**) Schematic cross section of Cordillera Zealandia showing the inferred under-thrust Gondwana margin converted to an eclogitic root following partial melting during a flare-up event, which produced the voluminous WFO and SPS suite magmatism.

Crustal profiles of the magmatic arc before and after c. 130 Ma (Fig. 3) were reconstructed by incorporating published seismic velocity and gravity geophysical data, Ce/Y ratios for mafic rocks^40^, Sr/Y ratios for intermediate rocks^41–43^ and maximum pressures recorded throughout the arc. Sr/Y data were filtered by MgO (1–6 wt%) and SiO_2_ (55–70 wt%) content, and then Sr/Y outliers were removed via the modified Thompson tau statistical method, similar to the methods described by Chapman *et al*.^41^. Median values were then obtained for the Darran Suite, Separation Point Suite and Western Fiordland Orthogneiss respectively.

### Steady-State (Pre-c.130 Ma: Darran Suite)

Hornblende-Al geobarometry records an emplacement depth of 15–22 km for the Darran Suite in Fiordland^29, 44^ providing a minimum estimate of the crustal thickness before c. 130 Ma. Utilizing the whole rock geochemical data of Allibone *et al.* ^21^, gabbroic rocks of the Darran Suite have maximum Ce/Y ratios of 2.5, equating to a crustal thickness in excess of 35 km^40^. The Darran Suite whole rock data also has median Sr/Y ratios of 28, equating to a crustal thickness of ~40 km^41, 42^. Seismic velocity data delineates the Moho at approximately 60 km depth beneath the whole of Fiordland^45^. However, it is unclear to what degree subsequent arc magmatism and the recent transpressional plate tectonic setting^46^ and subduction initiation modified the pre-c. 130 Ma crustal profile. Geochemical data are consistent with arc material older than c. 130 Ma having been emplaced in an arc approximately 40 km thick (Fig. 3).

### Flare-up (Post-c. 130 Ma: Separation Point Suite and Western Fiordland Orthogneiss)

The maximum-recorded emplacement depth of the Western Fiordland Orthogneiss is 18 kbar^47^, indicating a crustal thickness in excess of 60 km at c. 130 Ma, confirming early inferences of crustal thickness up to 70 km^48^. Utilizing the whole rock geochemical data of Allibone *et al.* ^22^, gabbroic rocks from these plutons have maximum Ce/Y ratios >5, extending the crustal thickness estimates for Fiordland of Mantle and Collins^40^ to greater than 60 km. Median Sr/Y ratios of 68 for the Western Fiordland Orthogneiss whole rock data set of Allibone *et al.* ^22^ estimate a thickness in excess of ~70 km^41, 42^. The Separation Point Suite’s median Sr/Y ratios of 108 fall outside the correlations established by Profeta *et al.* ^42^ and Chapman *et al.* ^41^ and may reflect higher degrees of differentiation^43^. Seismic velocity data extends the current surface geology to at least 10 km depth^45^. Higher velocity material, inferred by Eberhart-Phillips and Reyners^45^ to be dense residues/cumulates left behind in the source region of the Western Fiordland Orthogneiss, extends to more than 40 km depth where it is juxtaposed against the newly subducting Australian plate. These observations suggest that the crust may have been more than 80 km thick after c. 130 Ma (Fig. 3). Ducea^13^ and Ducea and Barton^6^ posit the residue/melt ratio in a batholith as 1/1 to 3/1. This implies that the 30 km of residues/cumulates imaged by Eberhart-Phillips and Reyners^45^ beneath the Western Fiordland Orthogneiss is smaller than expected for the predicted 80 km thick arc. The missing residue/cumulates may have either delaminated after flare-up or been tectonically removed during the recent transpressional plate tectonic setting and subduction initiation. The preservation of residue/cumulate materials^49–51^ is unusual as they commonly founder due to having densities greater than the mantle. The root of residue/cumulates may have been preserved by a buoyant subducting slab, and partially removed by recent subduction similar to the Laramides^13, 16^.

## New Magmatic Flux Rates and Isotopes

Richard Jongens (GNS Science) utilized a 1:250 000 digital map^20^ to calculate the outcrop area of each of the three volumetrically important Mesozoic components of the Median Batholith. The Darran Suite is exposed over approximately 2700 km^2^, the Separation Point Suite over approximately 1050 km^2^ and the Western Fiordland Orthogneiss Suite over approximately 2700 km^2^. The available geochronological data are consistent with the majority of the Darran Suite have been emplaced over approximately 40 my, and the Separation Point and Western Orthogneiss plutons having been emplaced coevally over approximately 10 my^21–23, 28, 31, 32, 36, 52^. The strike length of this segment of the arc is approximately 200 km.

To calculate the flux rate (km^3^/my per km of arc), the volume in km^3^ is estimated by utilising published data on crustal thickness (see Crustal Profile above, pre-130 Ma = 40 km thick, post-130 Ma => 60 km thick) and calculations of the area of the suites in km^2^ (see above). This is then divided by the length of the arc in the study (200 km) and further divided by the period for each suite emplacement (see above). This enables a direct comparison of flux rates estimated for the pre-c. 130 Ma Darran Suite and the combined post-c. 130 Ma Separation Point Suite and Western Fiordland Orthogneiss.

**Figure 4:**
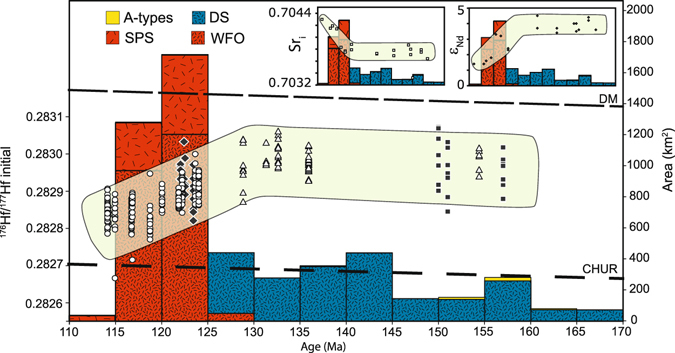
Hfi evolution over time for the three main Mesozoic plutonic suites of Fiordland combined with the flux rates (km^2^) over time. The excursion to evolved compositions is concomitant with the flare-up, attesting to the crustal component in these magmas. Circles: Western Fiordland Orthogneiss^28^, diamonds: Separation Point Suite and Western Fiordland Orthogneiss^24^), Triangles: this study, squares^29^. Inset of Sr_i_ and εNd^27, 32, 33^ over time (110–170 Ma) display complementing trends of involvement of an evolved crustal source post c. 130 Ma. See Fig. 1 for abbreviations, DM = depleted mantle, CHUR = Chondritic uniform reservoir.

The Darran Suite magmatic flux rate is 14 km^3^/my per km of arc. As the Separation Point Suite and Western Orthogneiss were coeval, their combined magmatic flux rate is greater than 100 km^3^/my per km of arc and possibly up to 150 km^3^/my per km of arc depending upon the thickness of the arc at the time. Individual plutons within these suites were assigned ages based on available geochronology or field relationships to create a histogram of age versus exposed area (Fig. 4).

Zircon grains from five rock samples of Darran Suite and their corellatives^25, 53, 54^ (Fig. 1), were previously dated via SHRIMP by Hollis *et al.* ^25^ and selected for Lu-Hf analyses via MC-LA-ICP-MS at Macquarie University, Sydney, Australia. See Supplementary Information for raw data and Milan *et al.* ^28^, for methods and operating conditions for Lu-Hf data collection.

The initial Hf isotopic ratio (Hf_i_) of the Darran Suite samples is consistent with recycling of a common ancient source with a c. 500 Ma average model age for all samples (this study, Scott *et al.* ^29^) during a 40 my period of low magmatic flux. The Hf_i_ of the Separation Point Suite^24^, and Western Fiordland Orthogneiss^28^ shows an excursion to less radiogenic values over time, during a brief pulse of high-flux magmatism. Sr_i_ and εNd data (Fig. 3 inset) from previous studies^27, 32, 33^ show an identical excursion towards an evolved component in the source region.

## Cyclicity in Cordilleran Arcs

Cordilleran arcs are characterized by cyclical trends in the flux and isotopic composition of the magma supplied to the upper plate^5^. The Mesozoic component of the Median Batholith in Fiordland, New Zealand has remarkable similarities to key features of one cycle identified in Cordilleran arcs of the Americas, including the coincidence of a high-flux event and excursions in both Sr/Y ratios^36^ and isotopic data. There is no evidence for a second Mesozoic high-flux event in Fiordland, suggesting that the Cordilleran cycle we have identified was the only cycle that took place there in the Mesozoic. The cycle halted when plate motions changed, leading to the collapse of Cordillera Zealandia and fragmentation of the margin at c. 105 Ma^55^. A paucity of exposed rock older than 170 Ma in the Median Batholith suggests that the arc had a low magmatic flux during this time period and precludes the possibility of a second cycle. Rare clasts of c. 200–180 Ma Median Batholith rocks in conglomerates^56^ have Ce/Y ratios consistent with the crust having been approximately 35 km thick at this time. These observations suggest the crust was 35–40 km thick from the Permian to c. 130 Ma. A good geological record in Fiordland exists from 170–114 Ma. The first ~40 my stage is characterised by low magmatic flux rates of the Darran Suite, consistent with dominantly mantle derived ‘background’ levels of arc magmatism^5, 12, 19^. The last 15 my stage is characterised by an increase of magmatic flux rates by one order of magnitude coupled with a significant excursion in Sr-Nd-Hf isotopic ratios and the modification of pre-existing arc crust through melt-rock interaction^54, 57, 58^. Flare-ups and isotopic excursions have been explained in the Americas via the underthrusting of a melt-fertile, lower crustal foreland into the base of the arc^5, 6^. In Fiordland, zircon inheritance age spectra in the Western Fiordland Orthogneiss are dominated by c. 2480, 770 and 555 Ma peaks which are consistent with the underthrusting or burial, and partial melting of an amalgam of foreland Gondwana margin crust^28^. The excursion to more evolved isotopic signatures, coupled with the high-flux event (Figs 3 and 4) attests to the importance of this crustal contribution to the magmatic flux.

## Arc Migration Over Time

Exhumed examples of long-lived Cordilleran arcs reveal they are constructed from a series of vertically aligned, trench-parallel igneous belts of varying age^7^. This wide footprint is produced by the migration of the active magmatic front inboard or outboard relative to a fixed position on the upper plate^7^. Inboard migrations are well documented in the American Cordilleras^7, 59, 60^, but outboard migrations in arcs also occur^7^.

For the Mesozoic Cordilleran Zealandia arc, the inboard migration of the active magmatic front (westward) during the flare up (Fig. 1) has been attributed to flat slab subduction^36^. The location of pre c. 130 Ma Darran Suite has led to competing tectonic interpretations. The most recent model (Scott *et al.* ^29^) calling for an allochthonous history of the Darran Suite and excision of a back-arc basin is based upon: (i) contrasting isotopic signatures either side of c. 130 Ma; (ii) the restriction of Gondwana-derived metasedimentary rocks to the west; (iii) a proposed crustal suture; and (iv) the presence of A-type granites. Here we argue against each of these points in turn and reaffirm an autochthonous relationship between the Darran Suite and western parts of the Mesozoic arc.

The apparent contrast in isotopic signatures are resolved in this study by new data defining a smooth excursion towards an evolved character, reflecting the underthrusting or burial of a Gondwanan lower crustal component, as required by the scale and geochemistry of the flare-up event. Though exposures of metasedimentary units in the proposed ‘allochthonous terrane’ lack Gondwana-derived detrital zircon, the very restricted nature of these units suggests that they represent small intra-arc basins formed during oblique subduction. In addition, significant Gondwana inheritance has been observed in Mesozoic plutons of the proposed ‘allochthonous terrane’ (e.g. Pomona Island and Clark Hut granites^21, 61^). The proposed crustal suture is unusually narrow (300 m wide), and lacks ophiolitic material that might support the argument for basin closure. The same structure has been interpreted (along strike) to reflect transpression within a Cordilleran setting^34^. Furthermore, intrusive relationships between the Western Fiordland Orthogneiss and parts of the Darran Suite are preserved in northern Fiordland^48, 62^.

Though Scott *et al.* ^29^ ascribe A-type granites in the Darran Suite to a rift setting, these are restricted to just ~42 km^2^ and a number of tectonic settings have been proposed for A-type granites elsewhere^63–66^. Furthermore, these rocks have similar Sr and Nd isotope ratios^32^ to the entire Darran Suite and intruded at a time of thickened crust inconsistent with a rift setting.

## Generation of Crust in Arcs

The flux from mantle to crust is basaltic (e.g. Davidson and Arculus^67^) in contrast to the andesitic composition of average continental crust^1, 68, 69^. The Fiordland flare-up event represented by the Separation Point Suite and Western Fiordland Orthogneiss equates to two thirds of the total volume of newly created arc crust throughout the Mesozoic in ‘Cordillera Zealandia’ and highlights the significance of short-lived high-flux episodes in Cordilleran arcs. Long-lived Cordilleran arcs of the Americas record repetitive flare-up contributions as high as 85–90% of the total volume of the arc^5–7, 12, 70–72^. These high-flux episodes require a significant component of the arc magma to be derived from melt-fertile lower crust. These observations diminish the contribution of mantle-derived melts in Cordilleran arcs and in addition to the foundering of dense residues/cumulates from the roots of arcs^13–16^, contribute to resolving why the average continental crust is andesitic in composition.

## Electronic supplementary material

Dataset Table A
